# Non-Structured Amino-Acid Impact on GH11 Differs from GH10 Xylanase

**DOI:** 10.1371/journal.pone.0045762

**Published:** 2012-09-21

**Authors:** Liangwei Liu, Xiaofeng Sun, Pengfei Yan, Linmin Wang, Hongge Chen

**Affiliations:** 1 Life Science College, Henan Agricultural University, Zhengzhou, Henan, China; 2 Key Laboratory of Enzyme Engineering of Agricultural Microbiology, Ministry of Agriculture, Zhengzhou, Henan, China; Russian Academy of Sciences, Institute for Biological Instrumentation, Russian Federation

## Abstract

The *Aspergillus niger* xylanase (Xyn) was used as a model to investigate impacts of un-structured residues on GH11 family enzyme, because the β-jelly roll structure has five residues (Ser1Ala2Gly3Ile4Asn5) at N-terminus and two residues (Ser183Ser184) at C-terminus that do not form to helix or strand. The N- or/and C-terminal residues were respectively deleted to construct three mutants. The optimal temperatures of XynΔN, XynΔC, and XynΔNC were 46, 50, and 46°C, and the thermostabilities were 15.7, 73.9, 15.5 min at 50°C, respectively, compared to 48°C and 33.9 min for the Xyn. After kinetic analysis, the substrate-binding affinities for birch-wood xylan decreased in the order XynΔC>Xyn>XynΔNC>XynΔN, while the K_cat_ values increased in the order XynΔC<XynΔNC<Xyn<XynΔN. The C-terminal deletion increased the GH11 xylanase thermostability and T_opt_, while the N- and NC-terminal deletions decreased its thermostability and optimal temperature. The C-terminal residues created more impact on enzyme thermal property, while the N-terminal residues created more impact on its catalytic efficiency and substrate-binding affinity. The impact of non-structured residues on GH11 xylanase was different from that of similar residues on GH10 xylanase, and the difference is attributed to structural difference between GH11 jelly-roll and GH10 (β/α)_8_.

## Introduction

Enzyme is widely used in industrial biocatalysis, such as fermentation, bio-energy production, food, beverage, paper and pulp, etc. However, the higher temperatures demand for robust enzymes. Many strategies have been used to improve enzyme thermostability. For example, disulphide-bonds were introduced to the *Trichoderma reesei* xylanase and the *Bacillus stearothermophilus* xylanase [1]–[3]. Homologous segments were recombined between two *Streptomyces lividans* xylanases [4]. The N-terminus of mesophilic *S. olivaceovirdis* xylanase was substituted with the homologous region of thermophilic *Thermomonospora fusca* xylanase [5]. Disulfide-bond was introduced to the *T. reesei* xylanase internal region and the *Thermomyces lanuginosus* xylanase N-terminus [6], [7].

From structural view point, enzyme is composed of irregular segment, α-helix, and β-strand. The later two are known as regular secondary structural elements. Unlike regular secondary structures, irregular segments are composed of amino-acids that are referred to as non-structured residues. Compared with stabilizing impact of regular secondary structures on enzyme thermostability [8], [9], impact of non-structured residue is seldom investigated. Recently, the non-structured residues were shown to disturb the *Aspergillus niger* GH10 xylanase thermostability and catalytic activity [10]. However, the GH10 xylanase exhibits a (β/α)_8_ structure, which represents only one family of enzymes, and the (β/α)_8_ structure consists mainly of α-helixes and β-strands [11]. The issue remains unsolved whether the non-structured residues have a general role or not in other family of enzymes. GH11 xylanase is a big family, and its β-jelly roll structure consists of 6% enzymes [12].

**Figure 1:**
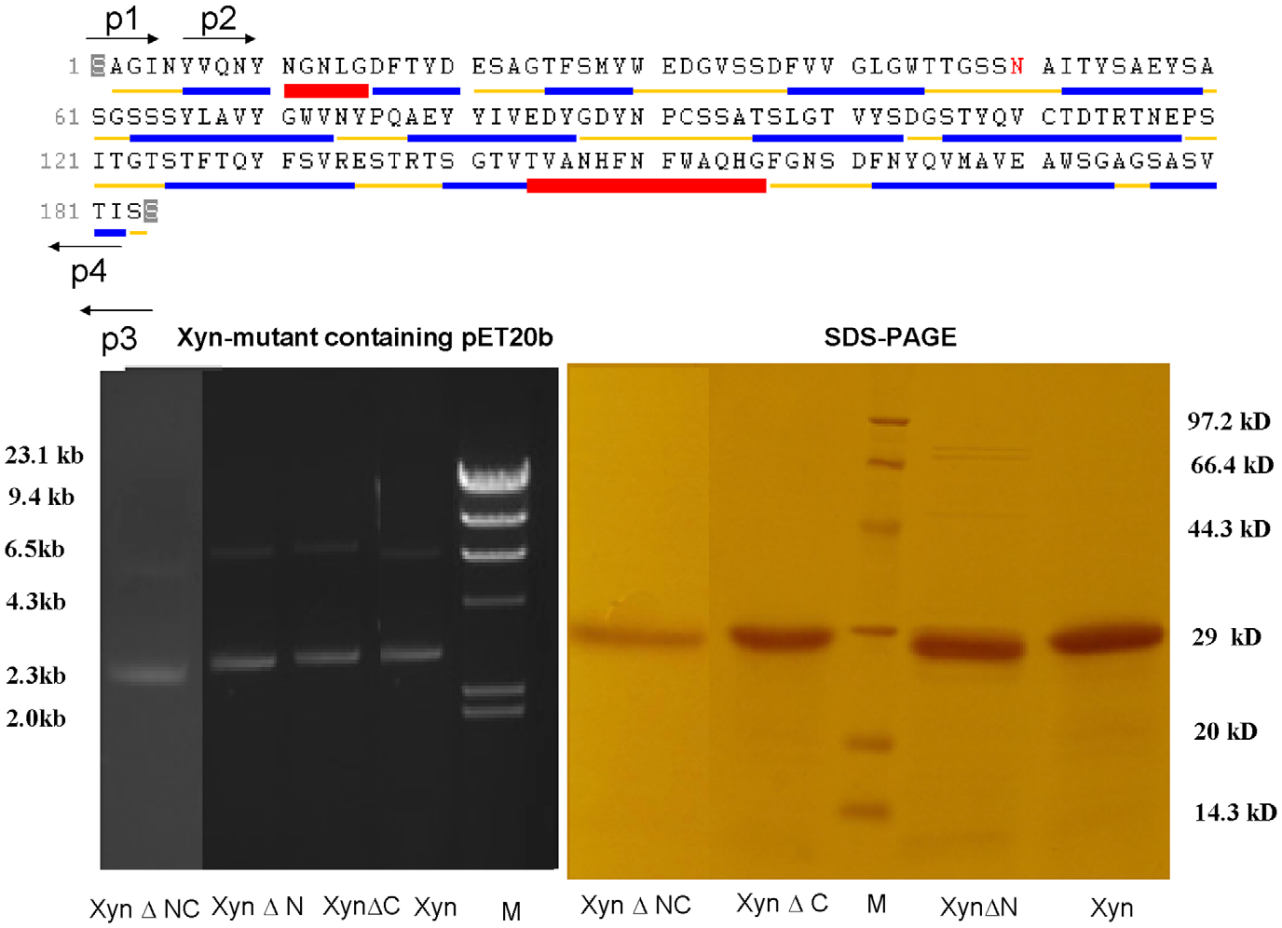
Construction of the deletion mutants. The *Aspergillus niger* Xyn secondary structural units (upper) were shown as irregular segment in thinner line (yellow), strand in middle line (blue), and helix in wider line (red). The genes, *Xyn*Δ*N*, *Xyn*Δ*C*, and *Xyn*Δ*NC* and *Xyn,* were amplified with primers p2/p3, p1/p4, p2/p4, and p1/p3, respectively. The recombinant pET20b(+) plasmids containing accurate genes appeared on the 1.4% gel as discrete bands at more than 2.3 kb (left), and the proteins appeared on the 12.5% SDS-PAGE as ∼29 kDa (right).

As a model of GH11 family enzyme, an xylanase (Xyn) was cloned from *A. niger* (GenBank: **EU375728**) [13]. The 185 amino-acid enzyme is the smallest GH11 xylanase, and exhibits the typical β-jelly roll structure (PDB: **1UKR**) [14], [15], unlike (β/α)_8_ structure of GH10 xylanase. The GH11 Xyn structure resembles a partially closed right hand, which is composed of two large β-pleated sheets and one helix [12]. The sheets are composed of anti- and parallel strands connected by irregular segments, and the only one helix is regarded to have stabilizing contribution to enzyme thermostability [8], [9]. Analyzed from structural level, the Xyn has five residues (Ser1Ala2Gly3Ile4Asn5) at N-terminus and two residues (Ser183Ser184) at C-terminus that do not form to helix or strand (Fig 1). The non-structured residue impacts on enzyme properties are investigated by respectively deleting the N- or/and C-terminal residues to construct three mutants, XynΔN, XynΔC, and XynΔNC. The deletions increase enzyme thermal properties and catalytic efficiencies. The study gives more insights into non-structured residue impacts on enzyme properties and provides more strategies for enzyme rational engineering.

## Results

### Construction of the Mutants

The XynΔN, XynΔC, and XynΔNC genes were amplified and cloned to the pET20b (Fig. 1). After sequence accuracies of the extracted recombinant plasmids had been confirmed by DNA sequencing, the transformed cells containing accurate recombinant plasmids, pET20b-*xyn*Δ*n*, pET20b-*xyn*Δ*c*, and pET20b-*xyn*Δ*nc*, were induced to express xylanases. The proteins, XynΔN, XynΔC, and XynΔNC, appeared on the SDS-PAGE gel as discrete bands at ∼29 kDa (Fig 1, Table 1). The larger apparent molecular masses are attributed to the xylanase having acidic property and a C-terminal His_6_ tag. The xylanase pI is 4.42, and its optimal reaction pH, pH_opt_, is 3.8. Acidic protein was found to bind less SDS, and therefore had a larger apparent molecular mass [17]–[19].

**Table 1:** Xylanase properties.

Xylanase	pH_opt_/pI	T_opt_ (°C)	t_1/2_ (50°C) (min)	V_max_(umol/l/s)/K_m_(mg/ml)/K_cat_(1/s)	MM(kDa) Apparent/Theoretical	Number ofamino acids
Xyn	3.8±0.3/4.42	48±0.1	33.9	14.6±2.8/14.6±4.4/200	29/21.1	184
XynΔN	3.6±0.3/4.42	46±0.2	15.7	16.1±3.9/38.8±11.7/222.7	28/20.6	179 (sagin)
XynΔC	3.6±0.2/4.42	50±0.3	73.9	10.6±0.6/6.6±0.8/158.9	28/20.9	182 (ss)
XynΔNC	3.6±0.2/4.42	46±0.2	15.5	12.2±1.0/25.2±2.8/182.0	28/20.4	177 (sagin)(ss)

Note: T_opt_: optimal reaction temperature for activity, t_1/2_: thermal in-activation half-life, after incubation at 50°C for different intervals, residual activity was determined and expressed as a ratio to the un-incubated enzyme, and data were fitted with Arrhenius function. The t_1/2_ was calculated according to the decay function to indicate enzyme thermostability. Using the Student’s t-Test (*p*<0.05), the XynΔC is more thermostable than the Xyn, and it is more thermostable than the XynΔN and the XynΔNC. The latter two enzymes are statistically similar thermostable. Kinetics was determined for birch-wood xylan at each enzyme pH_opt_ and T_opt_ conditions, and the data were fitted with Hill function to calculate V_max_ and K_m._

### Enzyme Properties

**Figure 2:**
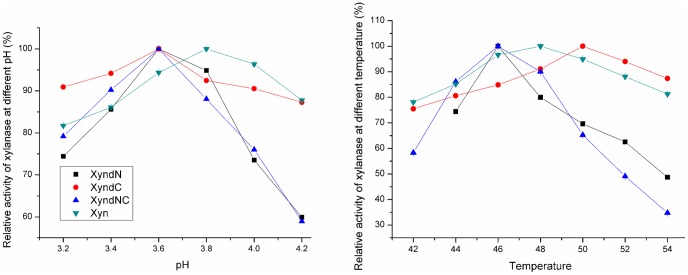
Xylanase optimal pH and optimal temperature. The pH_opt_ was determined from pH 3.2 to 4.2 at 0.2 unit interval in 50 mM imidazole-biphthalate buffer (left). The T_opt_ was determined from 42 to 54°C at 2°C interval (right).

**Figure 3:**
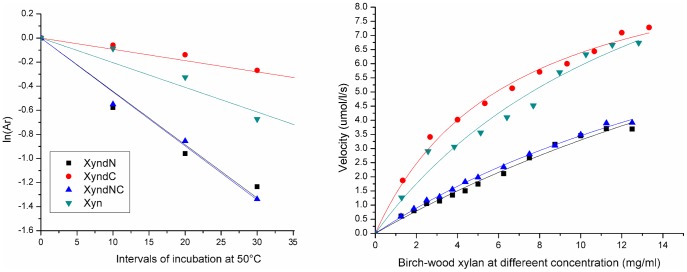
Xylanase thermostability and kinetics. After incubation at 50°C at 10-min interval from 10 to 30 min, the residual activity was assayed and expressed as a ratio relative to the un-incubated xylanase activity. The data were fitted with the equation y = A^*^e^-kt^ (Origin, version 8.0), and thermostability (t_1/2_) was calculated according to the decay function. The kinetics were assayed at T_opt_ and pH_opt_ conditions using birch-wood xylan at concentrations from 0 to 13 mg/ml. The data were fitted with the Hill function to calculate maximal activity (V_max_) and K_m_ (Origin, version 8.0).

The impacts of non-structured residues on enzyme properties were determined by assaying the mutants in parallel with the wild xylanase. Each data point was assayed for three independent reactions. The pH_opt_ values of three mutants were 3.6 in imidazole-biphthalate buffer, 0.2 units lower than the Xyn (Fig 2, Table 1). The optimal reaction temperatures, T_opt_ values, of XynΔN, XynΔC, and XynΔNC, were 46, 50, and 46°C, respectively, compared to 48°C for the Xyn (Fig 2, Table 1). Thus, the C-terminal residue deletion increased enzyme thermal activity, while the N- and NC-terminal residue deletions decreased its thermal activities. After incubation at 50°C, the residual activity were determined and fitted with the *Arrhenius* equation. The thermal in-activation half-lives (t_1/2_), thermostabilities, of XynΔN, XynΔC, and XynΔNC were calculated to be 15.7, 73.9, and 15.5 min, respectively. Both XynΔN and XynΔNC were **∼**50% thermostable as the Xyn; whereas, the XynΔC was 2.2-times more thermostable than the Xyn (Fig 3, Table 1). The C-terminal residue deletion increased enzyme thermostability, while the N- and NC-terminal residue deletions decreased enzyme thermostability. The increasing impact of C-terminal deletion on enzyme T_opt_ and thermostability was counteracted by the decreasing impact of N-terminal deletion, showing that the impact of N-terminal residues on enzyme thermal properties was bigger than that of the C-terminal residues.

When enzyme kinetics were determined at pH_opt_ and T_opt_ conditions, the substrate-binding affinities for birch-wood xylan decreased in the order XynΔC>Xyn>XynΔNC>XynΔN. The V_max_ values increased in the order XynΔC<XynΔNC<XynΔN, and the mutant V_max_ values were 70–110% that of the Xyn. The specific catalytic efficiencies (K_cat_) increased in the order XynΔC<XynΔNC<XynΔN, and the mutant K_cat_ values were 80–110% that of Xyn (Fig 3, Table 1). Unlike the decreasing impact of C-terminal deletion on enzyme activity, the N-terminal deletion increased enzyme catalytic efficiency, while the NC-terminal deletion created intermediate impact on enzyme catalytic efficiency. Thus, the increasing impact of N-terminal deletion on enzyme catalytic activity was counterbalanced by the decreasing impact of C-terminal deletion. The N-terminal deletion produced more impact on enzyme kinetic parameters.

### Structural Analysis

**Figure 4:**
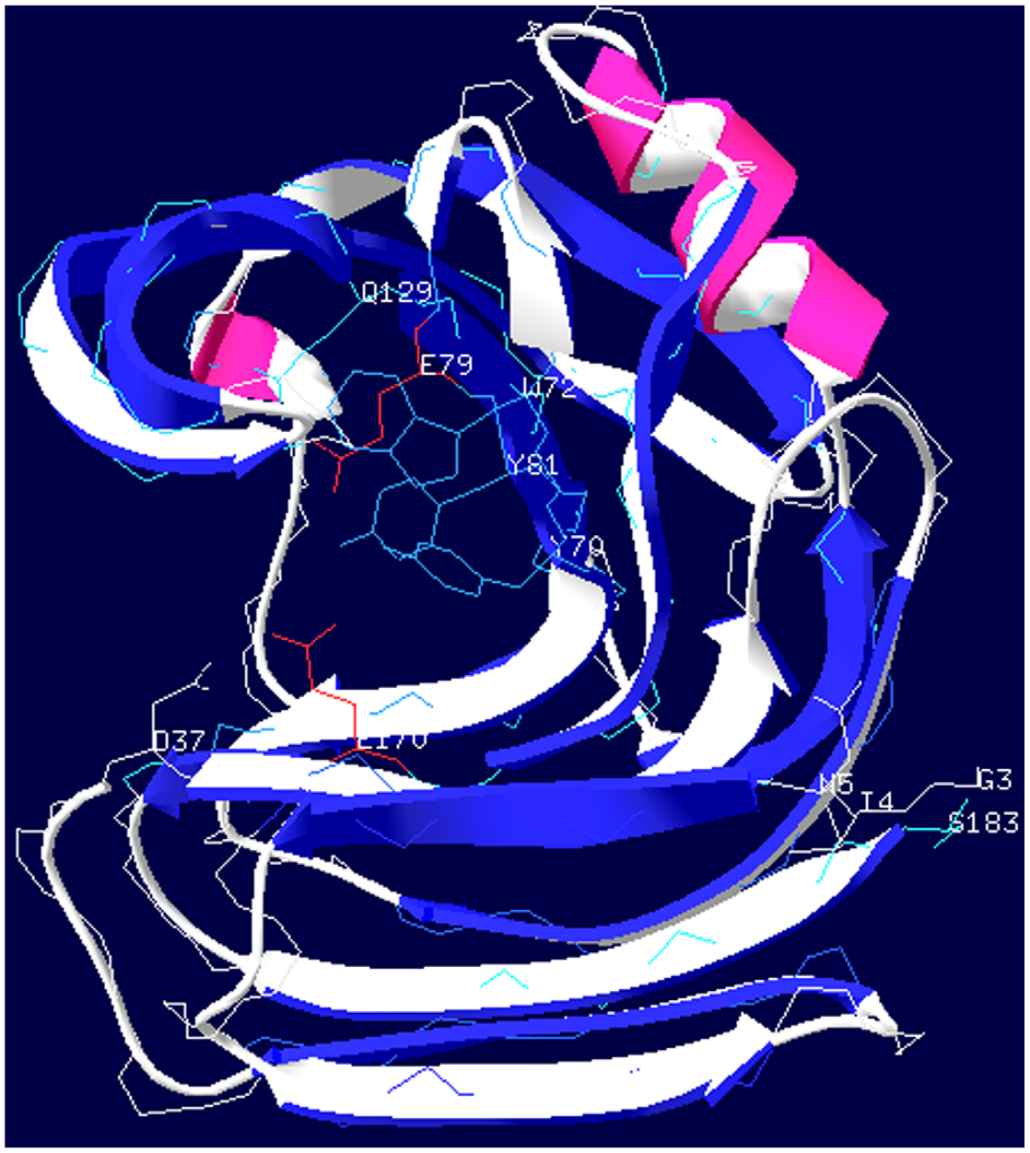
The *Aspergillus niger* Xyn structure. The N-terminal non-structured residues are Ser1Ala2Gly3Ile4Asn5, and the C-terminal non-structured residues are Ser183Ser184. Only the Gly3Ile4Asn5 and Ser183 were shown, because the deposited structure (PDB: **1UKR**) did not contain the Ser1Ala2 at N-terminus and the Ser184 at C-terminus. The active-site residues are Glu79 and Glu170. The substrate-binding residues are Asp37, Trp72, Tyr79, Tyr81, and Gln129. The N-terminal residues are closer to the substrate-binding residues, therefore, have more impact on enzyme catalytic activity and substrate-binding affinity. The C-terminal residues are more distal to the substrate-binding residues but closer to the C-terminus, therefore, have less impact on its catalytic properties but more impact on its thermal properties.

The five N-terminal non-structured residues created more impact on the enzyme substrate-binding affinity and catalytic activity, while the two C-terminal ones created more impact on its thermal properties. To explain the different impacts of N- and C-terminal residues on enzyme thermal and catalytic properties, the *A. niger* xylanase structure was drawn according to the PDB data (**1UKR**) (Fig 4) [14], [15]. Two active-site residues of the enzyme are Glu79 and Glu170. The nucleophile residue, Glu79, is held in place by interactions to Gln129 and Tyr70. The acid-base catalyst residue, Glu170, is hydrogen-bonded with Tyr81 and Asp37. According to the analysis of *A. niger* xylanase structure [14], substrate-binding sites of the Xyn are Gln129, Tyr70, Twr72, Tyr81, and Asp37. The N-terminal non-structured residues, Ser1Ala2Gly3Ile4Asn5, are closer to the substrate-binding residues, therefore, have more impact on enzyme kinetics. The C-terminal non-structured residues, Ser183Ser184, are more distal to the substrate-binding residues, but closer to the C-terminal strand, therefore, have less impact on enzyme activity, but more impact on thermal properties.

## Discussion

### Rational Deletion of Terminal Non-structured Residues

Previously, either the entire domains were truncated completely, or the terminal residues were deleted successively [20]–[26]. Unlike that, the GH11 xylanase seven terminal non-structured residues were rationally deleted based on structural analysis, because the residues do not form to regular secondary structural elements. The N-terminal deletion decreased enzyme T_opt_ for 2°C and thermostability for 50%, while the C-terminal deletion increased enzyme T_opt_ for 2°C and thermostability for 2.2-times. This is consistent with the homologous recombination analysis of *S. lividans* xylanase [4]. The deletion of N-terminal helix also increased the N-glycanase deglycosylation activity [22]. The successive truncation analysis showed that the seven N-terminal and the three C-terminal residues were unnecessary for the *Clostridium thermocellum* lichenase activity [23]. The five amino-acid mutations at N-terminus might confer structural stability, and hence, prevented the overall thermal unfolding of mesophilic *S. olivaceovirdis* xylanase [5]. The introduction of disulphide-bond showed that the flexible N-terminus disturbed enzyme thermostabilities [1]–[3], [6], [7]. The N- and C-terminal deletions created opposite effects on the enzyme thermal properties, thereby, the XynΔNC T_opt_ and thermostability decreased for 2°C and for 50%. However, the N-terminal deletion increased enzyme catalytic activity. Structural analysis shows that the N-terminal residues are closer to the substrate-binding residues, therefore, probably involve in substrate-binding.

### Different Impacts of Non-structured Residues between GH11 and GH10 Xylanase

Very recently, we investigated the impacts of similar non-structured residues on the GH10 xylanase. The N- or/and C-terminal deletions increased enzyme thermostability for ∼2 − 4-fold and activity for 4 − 7-fold [10]. The N-terminal deletion decreased the GH10 xylanase T_opt_ for 6°C, but the C-terminal deletion increased its T_opt_ for 6°C. The impacts of N- and C-terminal deletions were opposite on the enzyme T_opt_, but additive on its thermostability. The impact of five N-terminal residues was more on the GH10 enzyme kinetics, but less on its thermo-property than that of the one C-terminal residue. Compared with the two investigations, C-terminal deletion increased both GH10 and GH11 enzyme T_opt_ values and thermostabilities, and N-terminal deletion decreased both enzyme T_opt_ values. Whereas, N-terminal deletion decreased the GH11 enzyme thermostability, but increased the GH10 enzyme thermostability. The impacts of N- and C-terminal deletion were additive on GH10 enzyme thermostability, but opposite on GH11 enzyme thermostability. The inconsistence was attributed to the structural difference between GH10 (β/α)_8_ and GH11 β-jelly roll. The (β/α)_8_ structure is mainly composed of helixes and strands, with one (β/α) as a repeat unit; while the β-jelly roll structure is composed of many strands and only one helix, with one strand as a repeat unit. Unlike (β/α)_8_, β-jelly roll does not easily fold to active conformation. Moreover, the (β/α)_8_ structure is relatively more thermostable than the β-jelly roll. Probably, N-terminal deletion seriously disturbed the GH11 folding, but slightly disturbed the GH10 enzyme, because helix folds more easily than strand. The N-terminal non-structured residues are seated at upstream of strand, and probably, act as leading element for its folding. The C-terminal non-structured residues are seated at downstream of strand, therefore, scarcely disturb its folding.

At present, more and more structures were crystallized and deposited in PDB. As to those un-crystallized enzymes, structures can be modeled through comparative homologous modeling. All these progresses provide more structural information for enzyme rational engineering. According to the investigations of non-structured residue impact on GH11 and GH10 xylanases, N- and C-terminal non-structured residues are un-necessary for activities of both (β/α)_8_ and β-jelly roll enzymes. Strategy for rational enzyme engineering is proposed as that thermostability can be increased by deleting C-terminal non-structured residues, and catalytic efficiency can be increased by deleting N-terminal non-structured residues.

## Materials and Methods

### Materials and Reagents

The *A. niger* Xyn gene (GenBank: **EU375728**), which encodes a 185-residue mature xylanase, was cloned into pET20b(+) (Novagen, Shanghai, China). Molecular biology reagents, including *Pfu* polymerase, restriction endo-nucleases NdeI and XhoI, T4 DNA ligase, DNA marker, protein marker, were purchased from Takara Inc (Dalian, China).

### Construction of the Mutants

Based on structural analysis, the non-structured residues, Ser1Ala2Gly3Ile4Asn5 and Ser183Ser184, were respectively deleted to construct three mutants, XynΔN, XynΔC, and XynΔNC. PCR was carried out using 16.5 µg of pET20b-*xyn*, 1.0 µmol of each of two related primers, 5 U of *Pfu* polymerase, 4.0 µmol of dNTPs, and 1× polymerase buffer with the following thermal cycling: 4 min denaturation at 94°C, followed by 30 cycles of 1 min denaturation at 94°C, 1 min annealing at temperature given below, and 1 min extension at 72°C. The reaction was extended for 5 min at 72°C and cooled subsequently to 4°C.

The *Xyn*Δ*N* gene was amplified using p2/p3 and annealing at 33.6°C. The *Xyn*Δ*C* and *Xyn*Δ*NC* genes were amplified using p1/4 and p2/p4 and annealing at 35.8°C. The wild Xyn was amplified using p1/p3 and annealing at 37°C. The primer sequences used are as follows, with the underlined letters showing *Nde*I/*Xho*I restriction sites: p1: ggaattc*catatg*agtgccggtatca, p2: ggaattc*catatg*tacgtgcaaaactac, p3: ctaaatta*ctcgag*agaggagatcgtga; p4:ccaaatta*ctcgag*gatcgtgacactgg.

Following PCR amplification, the genes were cloned into pET20b(+) plasmids that had been pre-digested with NdeI/XhoI to delete redundant endo-nuclease sites. The recombinant plasmids were transformed into *E. coli* BL21(DE3) competent cells, then extracted and sequenced with an ABI 3730 automated sequencer to confirm gene accuracy (Invitrogen Biotechnology, Shanghai, China). The transformed cell containing accurate recombinant plasmids were grown and induced to produce xylanases according to standard protocols [16]. A C-terminal His_6_ tag was included in the xylanase sequences to allow the proteins to be purified with Co^2+^-binding resin (Amersham Bioscience). Active fractions were pooled and further purified using sephadex G-25. The xylanases were detected using 12.5% polyacrylamide SDS-PAGE, stained with Coomassie brilliant G-250. Protein concentration was measured by a Spectrophotometer ND-1000 at 280 nm using derivatization (NanoDrop Technologies, Wilmington, U.S.A).

### Enzyme Properties

The mutant enzyme properties were determined in parallel with the wild Xyn. All of the data points were determined for three independent reactions, and the averaged values were used. The optimal reaction pH, pH_opt_, was determined from pH 3.2 to 4.2 in 50 mM imidazole-biphthalate buffer at 0.2 unit interval. The optimal reaction temperature, T_opt_, was determined from 42 to 54°C at 2°C interval. The enzymes were pre-incubated at 50°C, and the residual activity was assayed at 10-min interval from 10 to 30 min and expressed as a ratio relative to the un-incubated xylanase activity. The data were fitted with the equation y = A*e^-kt^ (Origin, version 8.0), and the thermal in-activation half-life (t_1/2_) was calculated. The kinetics were assayed at T_opt_ and pH_opt_ conditions for 5 min using birch-wood xylan at concentrations from 0 to 13 mg/ml (Sigma-Aldrich, Shanghai, China). The data were fitted with the Hill function to calculate maximal activity (V_max_) and Km (Origin, version 8.0).

The xylanase standard activity was determined on birch-wood xylan using dinitrosalicylic acid method (DNS) [16]. Enzyme reaction was carried out for 16 min using 100 µL enzyme, 100 µL 1% birch-wood xylan, and 600 µL imidazole-biphthalate buffer, thereafter, 600 µL DNS was added and boiled. The reaction system was added to 5 mL with water, and absorbance was determined using UV2000 ultraviolet spectrophotometer at 550 nm.

## References

[1] Turunen O, Etuaho K, Fenel F, Vehmaanpera J, Wu X, et al (2001) A combination of weakly stabilizing mutations with a disulfide bridge in the alpha-helix region of *Trichoderma reesei* endo-1,4-beta-xylanase II increases the thermal stability through synergism. J Biotechnol 88: 37–46. 11377763 10.1016/s0168-1656(01)00253-x

[2] Fenel F, Leisola M, Janis JTO (2004) A de novo designed N-terminal disulphide bridge stabilizes the *Trichoderma reesei* endo-1,4-beta-xylanase II. J Biotechnol 108: 137–143. 15129722 10.1016/j.jbiotec.2003.11.002

[3] Jeong MY, Kim S, Yun CW, Choi YJ, Cho SG (2007) Engineering a de novo internal disulfide bridge to improve the thermal stability of xylanase from *Bacillus stearothermophilus* no. 236. J Biotechnol 127: 300–309. 16919348 10.1016/j.jbiotec.2006.07.005

[4] Wang Q, Xia T (2008) Importance of C-terminal region for thermostability of GH11 xylanase from *Streptomyces lividans*. Appl Biochem Biotechnol. 144: 273–282. 10.1007/s12010-007-8016-z 18556816

[5] Zhang S, Zhang K, Chen X, Chu X, Sun F, et al (2010) Five mutations in N-terminus confer thermostability on mesophilic xylanase. Biochem Biophys Res Commun 395: 200–206. 20361933 10.1016/j.bbrc.2010.03.159

[6] Xiong H, Fenel F, Leisola M, Turunen O (2004) Engineering the thermostability of *Trichoderma reesei* endo-1,4-beta-xylanase II by combination of disulphide bridges. Extremophiles 8: 393–400. 15278768 10.1007/s00792-004-0400-9

[7] Wang Y, Fu Z, Huang H, Zhang H, Yao B, et al (2012) Improved thermal performance of *Thermomyces lanuginosus* GH11 xylanase by engineering of an N-terminal disulde bridge. Bioresour. Technol. 112: 275–279. 10.1016/j.biortech.2012.02.092 22425398

[8] Hakulinen N, Turunen O, Janis J, Leisola M, Rouvinen J (2003) Three-dimensional structures of thermophilic beta-1,4-xylanases from *Chaetomium thermophilum* and *nonomuraea flexuosa*. comparison of twelve xylanases in relation to their thermal stability. Eur J Biochem 270(7): 1399–1412. 12653995 10.1046/j.1432-1033.2003.03496.x

[9] Liu L, Chen H, Jia X (2008) The influence of secondary structure content on thermostability of F/10 xylanase. J Biotechnol 136s: s201–s201.

[10] Liu L, Zhang G, Zhang Z, Wang S, Chen H (2011) Terminal amino-acids disturb xylanase thermostability and activity. J Biol Chem 286: 44710–44715. 22072708 10.1074/jbc.M111.269753 PMC3247970

[11] Leggio L, Kalogiannis S, Bhat M, Pickersgill R (1999) High resolution structure and sequence of *T.aurantiacus* xylanase I: Implications for the evolution of thermostability in family 10 xylanases and enzymes with beta alpha barrel architecture. Proteins 36: 295–306. 10409823

[12] Torronen A, Rouvinen J (1997) Structural and functional properties of low molecular weight endo-1,4-b-xylanases. J Biotechnol. 57: 137–149. 10.1016/s0168-1656(97)00095-3 9335170

[13] Chen H, Yan X, Liu X, Wang M, Huang H, et al (2006) Purification and characterization of a novel bifunctional xylanase, XynIII, isolated from *Aspergillus niger* A-25. J Microb Biot 16: 1132–1138.

[14] Krengel U (1996) Three-dimensional structure of endo-1,4-xylanase I from *Aspergillus niger*: Molecular basis for its low pH optimum. J Mol Biol 263: 70–78. 8890913 10.1006/jmbi.1996.0556

[15] Krengel U, Rozeboom H, Kalk K, Dijkstra B (1996) Crystallization and preliminary crystallographic analysis of endo-1,4-beta-xyalanase I from *Aspergillus niger.* Acta Crystallogr. Sect.D. 52: 571–576. 10.1107/S0907444995014740 15299682

[16] Liu L, Cheng J, Chen H, Li X, Wang S, et al (2011) Directed evolution of a mesophilic fungal xylanase by fusion of a thermophilic bacterial carbohydrate-binding module. Process Biochem 46: 395–398.

[17] Kaufmann E, Geisler N, Weber K (1984) SDS-PAGE strongly overestimates the molecular masses of the neurofilament proteins. FEBS Lett 170: 81–84. 6723964 10.1016/0014-5793(84)81373-3

[18] Millward-Sadler SJ, Davidson K, Hazlewood GP, Black GW, Gilbert HJ, et al (1995) Novel cellulose-binding domains, NodB homologues and conserved modular architecture in xylanases from the aerobic soil bacteria *Pseudomonas fluorescens* subsp. *cellulosa* and *cellvibrio* mixtus. Biochem J 312: 39–48. 7492333 10.1042/bj3120039 PMC1136224

[19] Wassenberg D, Schurig H, Liebl W, Jaenicke R (1997) Xylanase XynA from the hyperthermophilic bacterium *Thermotoga maritima*: Structure and stability of the recombinant enzyme and its isolated cellulose-binding domain. Protein Sci 6: 1718–1726. 9260284 10.1002/pro.5560060812 PMC2143759

[20] Huang B, Yu B, Rogers J, Byeon I, Sekar K, et al (1996) Phospholipase A2 engineering. deletion of the C-terminus segment changes substrate specificity and uncouples calcium and substrate binding at the zwitterionic interface. Biochemistry 35: 12164–12174. 8810924 10.1021/bi960234o

[21] Wen T, Chen J, Lee S, Yang N, Shyur L (2005) A truncated *Fibrobacter succinogenes*1,3–1,4-β-d-glucanase with improved enzymatic activity and thermotolerance. Biochemistry 44: 9197–9205. 15966744 10.1021/bi0500630

[22] Wang S, Xin F, Liu X, Wang Y, An Z, et al (2009) N-terminal deletion of peptide: N-glycanase results in enhanced deglycosylation activity. PLoS ONE 4: 1–8. 10.1371/journal.pone.0008335 PMC2791212 20016784

[23] Niu D, Zhou X, Yuan T, Lin Z, Ruan H, et al (2010) Effect of the C-terminal domains and terminal residues of catalytic domain on enzymatic activity and thermostability of lichenase from *Clostridium thermocellum* . Biotechnol Lett 32: 963–967. 20229062 10.1007/s10529-010-0241-9

[24] Hamana H, Shinozawa T (1999) Effects of C-terminal deletion on the activity and thermostability of orotate phosphoribosyltransferase from *Thermus thermophilus.* . J Biochem 125: 109–114. 9880805 10.1093/oxfordjournals.jbchem.a022246

[25] Ma Y, Eglinton J, Evans D, Logue S, Langridge P (2000) Removal of the four C-terminal glycine-rich repeats enhances the thermostability and substrate binding affinity of barley beta-amylase. Biochemistry 39: 13350–13355. 11063571 10.1021/bi000688s

[26] Phannachet K, Raksat P, Limvuttegrijeerat T, Promdonkoy B (2010) Production and characterization of N- and C-terminally truncated Mtx2: A mosquitocidal toxin from *Bacillus sphaericus* . Curr Microbiol 61: 549–553. 20411263 10.1007/s00284-010-9651-0

